# Pneumovirus in Dogs with Acute Respiratory Disease

**DOI:** 10.3201/eid1606.091778

**Published:** 2010-06

**Authors:** Randall W. Renshaw, Nancy C. Zylich, Melissa A. Laverack, Amy L. Glaser, Edward J. Dubovi

**Affiliations:** Cornell University, Ithaca, New York, USA

**Keywords:** Paramyxoviridae, pneumovirinae, respiratory syncytial virus, murine pneumovirus, canine respiratory disease, viruses, dispatch, Suggested citation: Renshaw RW, Zylich NC, Laverack MA, Glaser AL, Dubovi EJ. Pneumovirus in dogs with acute respiratory disease. Emerg Infect Dis [serial on the Internet]. 2010 Jun [date cited]. Available from http://www.cdc.gov/EID/content/16/6/993.htm

## Abstract

To determine which respiratory viruses circulate among confined dogs, we analyzed nasal and pharyngeal swab specimens from shelter dogs with acute respiratory disease. An unknown virus was isolated. Monoclonal antibody testing indicated that it was probably a pneumovirus. PCR and sequence analysis indicated that it was closely related to murine pneumovirus.

Domestic dogs housed in close confinement, as in kennels or animal shelters, are often involved in outbreaks of acute respiratory disease (*1*,*2*). To determine which viruses are associated with these outbreaks, we studied 200 dogs in 2 animal shelters in the northeastern United States during 2008–2009.

## The Study

Nasal and pharyngeal swab specimens were collected from the dogs, and swab eluate extracts were prepared. Pooled extracts were added to monolayer cultures of canine A72 cells (American Type Culture Collection, CRL-1542). After 3 passages in culture, cells in some of the flasks showed subtle cytopathic changes. After continued passage, small foci of rounded cells developed, and rapid and progressive cell death throughout the flask ensued in a pattern uncharacteristic of the viruses commonly isolated from dogs. Testing these cultures with a panel of diagnostic reagents specific for common canine respiratory agents failed to identify a known virus. Immunofluorescence assays (IFAs) ultimately detected 13 positive cultures over a 4-month period when a monoclonal antibody (MAb) pool against human respiratory syncytial virus (RSV) (VP-R151, Vector Laboratories, Burlingame, CA, USA) was used. We have commonly used this antibody preparation to detect bovine RSV. The staining pattern included filamentous membrane-bound and free-floating virions and cytoplasmic inclusions, typical of the pattern in RSV-infected cells.

After obtaining IFA results, we attempted to amplify a fragment of the nucleocapsid gene (N) from the virus by using PCR primers designed on the basis of an alignment of human, bovine, and ovine RSV sequences. This attempt was unsuccessful (data not shown). Stocks of the individual MAbs in the anti-RSV pool and their specificities were obtained from the manufacturer and used for IFA (Figure). MAb 2G122 (P protein–specific) stained primarily inclusions and gave a relatively uniform membrane-associated signal. Staining with MAb 5H5N (M2 protein–specific) illuminated virions and inclusions. No fluorescence was noted with MAbs 1C3 (N protein–specific) or 5A6 (F protein–specific). All 4 individual MAbs recognized bovine RSV by IFA (data not shown). Recognition of the canine virus by only 2 of 4 MAbs and the inability to amplify a conserved region of the RSV genome suggested that it was related to, but unlikely to be, RSV.

**Figure:**
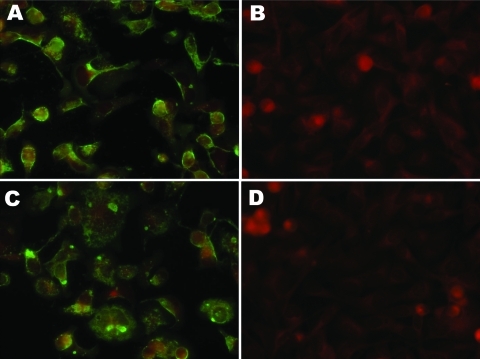
Slides showing immunofluorescence of A72 cells with human respiratory syncytial virus monoclonal antibodies (MAbs). A) MAb 2G122 on infected cells. B) MAb 2G122 on uninfected cells. C) MAb 5H5N on infected cells. D) MAb 5H5N on uninfected cells. Primary MAb stocks were used as obtained from the manufacturer at a dilution of 1:100. The red background is produced by counterstaining with Evans blue dye. Original magnification ×200.

To elucidate sequence information from 1 of the 13 isolates, we used a consensus-degenerate hybrid oligonucleotide primer algorithm to design degenerate PCR primers based on highly conserved amino acid sequences within multiple sequence alignments of all viruses in the subfamily *Pneumovirinae* (*3*). Specific regions within the L (polymerase) and N genes were targeted (Table). Sequencing of the reaction products and BLAST (www.ncbi.nlm.nih.gov/blast/Blast.cgi) analysis showed the virus to be closely related to murine pneumovirus (MPV), traditionally known as pneumonia virus of mice. Two L-gene PCR products were found to be 96%–97% identical to MPV, and an N gene fragment was 96% identical (Table).

**Table:** Partial gene locations of virus isolated from dogs with respiratory disease and relatedness to other pneumoviruses*

Region sequenced†	PCR primers (5′ → 3′) and aa target sequences in pneumoviruses	% Identity to MPV, nt, aa†	% Identity to HRSV, nt, aa‡	% Identity to HMPV, nt, aa§
N gene,¶ nt 852–1182 (331 bp)	P1R: ggaactcgggggcgaayttytccat target: MEKFAPEFH N276F: tccgtgcaggccgaratggarcarg target: SVQAEMEQV	96.4, 96.3	68.5, 70.1	56.5, 54.1
L gene, no. 1,¶ nt 1143–1452 (310 bp)	L428F: ccggatcttcggccayccnatggt target: RIFGHPMV L538R: ttcttaggaggggagatggcyttrtcrtt target: NDKAISPPKN	95.8, 97.1	62.1, 55.3	58.9, 47.6
L gene, no. 2,¶ nt 1962–2511 (550 bp)	L698F: catcaccgacctgtccaagttyaaycargc target: ITDLSKFNQA L894R: ttgaagtcgtccaggatggtrttdatcca target: WINTILDDFK	96.7, 99.5	68.4, 71.6	66.4, 68.3

*MPV, murine pneumovirus; nt, nucleotide; aa, amino acid; HRSV, human respiratory syncytial virus; HMPV, human metapneumovirus; N, nucleocapsid; R, reverse primer; F, forward primer L, polymerase. †Based on comparison with MPV strain J3666, GenBank accession no. NC_006579. ‡Based on comparison with HRSV, GenBank accession no. NC_001803. §Based on comparison with HMPV, GenBank accession no. NC_004148. ¶The N gene fragment is entered under GenBank accession no. GU247050; L gene fragments nos. 1 and 2 are entered under GenBank accession no. GU247051.

## Discussion and Conclusions

MPV is 1 of only 3 virus species classified in the family *Paramyxoviridae*, subfamily *Pneumovirinae,* genus *Pneumovirus.* Human RSV is the type species and is closely related to bovine RSV; MPV is more distantly related. For example, the N protein nucleotide sequences of human and bovine RSV are ≈94% identical to each other but only 60% identical to those of MPV. Only 2 strains of MPV, J3666 and Strain 15, have been fully sequenced, and they are 99.7% identical at the nucleotide level.

The nucleotide identity between virus isolated from the dogs and MPV was consistently >95% throughout the gene regions examined. Because the degenerate primers used were designed to cover regions that are generally highly conserved, regions of the genome that are typically more variable in other pneumoviruses may show greater differences between the canine pneumovirus and MPV. Of the conserved regions sequenced, amino acid identities were 70% (N), 55% (L fragment no. 1), and 72% (L fragment no. 2) when aligned with the same regions of human RSV, so finding cross-reactivity between human RSV MAbs and the newly isolated virus is not completely unexpected. Serologic cross-reactivity between RSV and MPV has been previously observed. Gimenez et al. (*4*) did not find recognition of MPV when they used 2 anti–human RSV MAbs, but they did observe recognition of MPV N protein when they used mouse anti–human RSV serum in immunoblot assays. Ling and Pringle (*5*) showed cross-reactivity of N proteins with polyclonal serum and cross-reactivity with a MAb that recognized P in immunoblot assays. Although the MAbs used in this study had been extensively tested for reactivity against a panel of 13 viruses, including several members of the family *Paramyxoviridae* (*6*,*7*), they were probably not tested for cross-reactivity against MPV because it is not a known human pathogen. The finding that reagents used to identify human RSV showed strong recognition of a closely related but distinct virus highlights the need for caution when interpreting research studies and conducting diagnostic evaluations.

The isolation of a previously unknown virus from dogs does not imply disease causation. However, comparison with MPV leads to speculation that the virus isolated in this study may have pathogenic potential. MPV is commonly known to infect laboratory rodent colonies, and serologic evidence points to infection of several wild rodent species. However, little is known about MPV epidemiology, such as whether MPV has multiple natural hosts or whether closely related viruses are circulating in other species. Neutralizing antibodies to MPV in other mammalian species, including humans, were first reported in the 1940s (*8*,*9*), and more recently, high prevalence of neutralizing antibodies but low (3%–4%) association with clinical disease in humans has been reported (*10*). Natural infection of rodents may be subclinical or latent. Sequelae to experimental infection in laboratory mice can vary from asymptomatic to severe disease with high morbidity and mortality rates. Pathogenic strains, including J3666 and Strain 15, can produce severe pneumonia and death in 6–10 days after mouse inoculation with a low dose (*11*,*12*). The pathogenicity or lack thereof may depend on the virus and on the mouse strain (*13*).

Questions remain as to whether this newly isolated virus commonly infects dogs and, if so, why it has not been previously isolated. Perhaps the strain that was circulating in these particular animal shelters is more easily isolated in culture. Or, because the initial cytopathic changes observed with these isolates were subtle, they could easily have been missed. Outbreaks of acute respiratory disease in dogs often involve multiple pathogens. As anticipated, other viruses, primarily canine influenza and parainfluenza viruses, were isolated during the study, often from the same animals that carried the pneumovirus. Work is ongoing to further determine pneumovirus prevalence among dogs and its involvement in acute respiratory disease of dogs.
